# Role of Recently Migrated Monocytes in Cigarette Smoke-Induced Lung Inflammation in Different Strain of Mice

**DOI:** 10.1371/journal.pone.0072975

**Published:** 2013-09-13

**Authors:** Sandra Pérez-Rial, Laura del Puerto-Nevado, Raúl Terrón-Expósito, Álvaro Girón-Martínez, Nicolás González-Mangado, Germán Peces-Barba

**Affiliations:** Respiratory Research Group, Instituto de Investigación Sanitaria-Fundación Jiménez Díaz-CIBERES (IIS-FJD-CIBERES), Madrid, Spain; University of Giessen Lung Center, Germany

## Abstract

This study investigates the role of proinflammatory monocytes recruited from blood circulation and recovered in bronchoalveolar lavage (BAL) fluid in mediating the lung damage in a model of acute cigarette smoke (CS)-induced lung inflammation in two strains of mice with different susceptibility to develop emphysema (susceptible -C57BL/6J and non susceptible -129S2/SvHsd). Exposure to whole-body CS for 3 consecutive research cigarettes in one single day induced acute inflammation in the lung of mice. Analysis of BAL fluid showed more influx of recently migrated monocytes at 72 h after CS-exposition in susceptible compared to non susceptible mice. It correlated with an increase in MMP-12 and TNF-α protein levels in the lung tissue, and with an increment of NF-κB translocation to the nucleus measured by electrophoretic mobility shift assay in C57BL/6J mice. To determine the functional role of these proinflammatory monocytes in mediating CS-induced airway inflammation, alveolar macrophages and blood monocytes were transiently removed by pretreatment with intratracheal and intravenous liposome-encapsulated CL_2_MDP, given 2 and 4 days prior to CS exposure and their repopulation was studied. Monocytes/macrophages were maximally depleted 48 h after last liposome application and subsequently recently migrated monocytes reappeared in BAL fluid of susceptible mice at 72 h after CS exposure. Recently migrated monocytes influx to the lung correlated with an increase in the MMP-12 protein level in the lung tissue, indicating that the increase in proinflammatory monocytes is associated with a major tissue damaging. Therefore our data confirm that the recruitment of proinflammatory recently migrated monocytes from the blood are responsible for the increase in MMP-12 and has an important role in the pathogenesis of lung disease induced by acute lung inflammation. These results could contribute to understanding the different susceptibility to CS of these strains of mice.

## Introduction

Cigarette smoke (CS) is the leading cause of chronic obstructive pulmonary disease (COPD) characterized by an abnormal persistent inflammatory response. Although >90% of patients with COPD are smokers, only a minority (±15–20%) of susceptible tobacco smokers have been reported to develop clinically significant COPD and the reason for this is unknown. The effect of CS in mice is believed to be strain dependent. However, the molecular basis of susceptibility of mouse strains to effects of CS is not known. Previous studies have demonstrated that C57BL/6J mice responded to CS exposure with accelerated development of emphysema [1], [2], [3], [4], [5] while those of the strain 129S2/SvHsd, which produce low levels of tumor necrosis factor-alpha (TNF-α), were resistant to lung inflammation and oxidant responses to CS exposure [4], [5] showing no inflammatory response to smoke at 24 h [6].

CS induces an exaggerated influx of inflammatory cells from the blood circulation into the airways, being these cells accessible through the bronchoalveolar lavage (BAL) fluid. Among all the inflammatory cells, alveolar macrophages play a pivotal role in the pathogenesis of COPD. Blood monocytes are well-characterized precursors for macrophages but alveolar macrophages turnover rate is slow and is maintained by constitutively immigrating resident monocytes [7], [8], [9]. In contrast, proinflammatory monocytes rapidly migrate into alveolar airspaces after lung infection and are believed to be the main effectors of acute lung injury [10], [11]. However, the limited characterization of the murine monocytes in BAL fluid has made difficult to identify the monocytes recruitment to inflammatory sites and may have led to an underestimation of their early migration [12].

Macrophages release a number of matrix metalloproteinases (MMPs) such as MMP-12, with potential degrading activity on lung matrix and the production of this protease has been found to be elevated in patients with COPD [13], [14]. The inflammatory properties for MMP-12 are linked to its capacity to release TNF-α from macrophages [15]. It is known that TNF-α drive 70% of CS-induced emphysema in the mouse [16]. Furthermore, free radicals, derived from cigarette smoke, activate the transcription of nuclear factor-kappa-light-chain-enhancer of activated B cells (NF-κB) [17], which in turn leads to the expression of many genes which encode mediators of the inflammatory process.

To determine the functional role of proinflammatory recently migrated monocytes in mediating acute CS-induced airway inflammation, one of the proposal methods was selectively and transiently deplete alveolar macrophages and blood monocytes using a well-established liposome-encapsulated dichloromethylene diphosphonate (CL_2_MDP) strategy [18], [19], [20], [21] and subsequently their repopulation after CS exposure was studied.

The goal of the present study is to determine the role of proinflammatory recently migrated monocytes in acute CS-induced airway inflammation in two strains of mice with different susceptibility to develop emphysema. For this purpose, alveolar macrophages and blood monocytes were transiently depleted by administration of liposome-encapsulated CL_2_MDP, in order to assess the influx of renewed monocytes after CS exposure. Furthermore, we investigated whether MMPs are released in response to monocyte activation. The main findings are that proinflammatory monocytes are responsible directly in mediating CS-induced lung inflammation and that this cells release the MMPs in response to lung inflammation.

## Materials and Methods

### Animals

One hundred twelve adult 12-week-old males mice (22–25 g body wt) belonging to one of two strains -susceptible (C57BL/6J, Charles River Laboratories) and non susceptible (129S2/SvHsd, Harlan Iberica) to smoking-induced emphysema were housed in the Inhalation Core Facility at IIS-Fundación Jiménez Díaz for one week of acclimatization before the experiment. After that, the mice were returned to their cages, where a diet of alfalfa-free rodent food (Harlan Teklad) and water were provided *ad libitum*. The studies described herein were performed under a project license issued by the Ministry of Innovation and Science of Spain and all protocols were approved by the local Ethical Animal Research Committee at IIS-Fundación Jiménez Díaz. In all cases, the legislation regarding animal treatment, protection and handling was followed (RD 53/2013).

### Cigarette smoke (CS)-induced inflammation methodology

Experimental groups consisted of eight mice each. Cigarette smoke (CS) exposure was performed in a single exposition using smoke from 3 consecutive (with 5-minute breaks between them) standard research non filtered cigarettes (2R1, University of Kentucky, Lexington, KY, USA; 11.7 mg TPM (total particulate matter per cubic meter of air), 9.7 mg tar and 0.85 mg nicotine per cigarette). Mainstream CS was generated by an exposure system where combustion of the cigarette was drawn into the mice chambers via a peristaltic pump (KD Scientific, Inc.) [22] with some modifications [23]. Research cigarettes were smoked according to the Federal Trade Commission protocol (1puff/min of 2 seconds' duration and 35 mL volume) with fresh air being pumped in for the remaining time. Non smoke-exposed mice were administered filtered air in an identical chamber according to the protocol described for CS exposure.

### Measurement of carboxyhemoglobin (COHb) levels in blood samples

Immediately after removal of the animals from the smoke chamber, blood samples (∼50 µl) were collected in a heparinised tube by puncturing the retro-orbital sinus. COHb levels were measured spectrophotometrically with an IL 682™ CO-Oximeter (Instrumentation Laboratory) in order to confirm a non toxic exposure.

### Monocyte/macrophage transient depletion with liposome-encapsulated Cl_2_MDP

Liposome-encapsulated Cl_2_MDP (dichloromethylene diphosphonate) and liposome-encapsulated PBS were prepared as described [21]. Cl_2_MDP is a not toxic drug that once delivered into phagocytic cells using liposomes as vehicles, damage the cells irreversibly and die by apoptosis [24], [25]. Liposomes have a nearly unhindered access to these monocytes/macrophages as concluded from their fast but transient depletion, within 1 day liposome-encapsulated CL_2_MDP treatment in mice and rats [26] and persists for up to 5 days [27]. At day 5 after administration, some alveolar macrophages could be found returning, but complete repopulation of the lung with alveolar macrophages was only reached around day 18 [27]. Free Cl_2_MDP, e.g. released from dead macrophages, has an extremely short half life in the circulation and removed by the renal system. Moreover it is known that alveolar macrophage elimination *in vivo* is associated with an increase in pulmonary immune response in mice [27], [28]. Two consecutive injections with a time interval of 2 days were required to get a nearly complete depletion of macrophages and monocytes. Depletion is transient since cells are renewed from bone marrow precursors. In contrast, interstitial macrophages that are separated from the alveolar space by an epithelial barrier remain unaffected by the depletion procedure.

Mice received suspension of liposome-encapsulated CL_2_MDP in PBS with a final concentration of 5 mg/ml, at an effective dose of 200 µl/mouse injected intravenously (i.v.) and 100 µl/mouse intratracheal (i.t.) instillation twice, 4 and 2 days before CS exposure, as predetermined by preliminary studies. Control mice received empty liposomes without clodronate suspended in PBS (100 µl/mouse).

### Determination of BAL fluid leukocyte population by flow cytometry analysis

In order to perform BAL fluid, the mice were sacrificed with an overdose of sodium pentobarbitone (200 mg i.p.; Abbott Laboratories) and a tracheal cannula (22GA) was inserted to instill 1 ml of cold 0.9%NaCl and recover it by gentle manual aspiration. Cell viability was determined manually by using trypan blue exclusion. The absolute number of inflammatory cells in the recovered BAL fluid was determined by counting on a hemocytometer (cells×10^3^/ml). The remaining BAL fluid cells were used to flow cytometry analysis. Immunostaining was performed using fluorochrome-conjugated monoclonal anti-mouse macrophage-specific F4/80-Phycoerythrin (PE) and neutrophil-specific Ly6B.2 (clone 7/4)-AlexaFluor647 (AF647) antibodies (both from Serotec Ltd) directed at leukocytes surface markers. BAL fluid suspension (0.5–1×10^6^ cells approx.) was treated with CD16/CD32 (clone 2.4G2) antibody (BD Biosciences) to block Fcγ II/III receptors and reduce nonspecific binding. The samples were stained and washed before data acquisition on a dual-laser FACS Calibur™ flow cytometer running CELL Quest™ software (BD Biosciences) using an absolute counting protocol. List mode data were analyzed with Infinicyt™ software (Cytognos). BAL fluid cells were identified based on forward and side scatter characteristics (FSC and SSC): neutrophils (F4/80^−^ Ly6B.2^hi^), recently migrated monocytes (F4/80^+^ Ly6B.2^+^) and recently differentiated alveolar macrophages (F4/80^hi^ Ly6B.2^lo^).

### Lung tissue and protein isolation

Lung tissue samples were obtained from sacrificed animals at 24, 48 and 72 h after CL_2_MDP treatment and CS exposure. Protein extraction from lung tissue was performed with the NE-PER™ commercial kit according to the manufacturer's instructions (Thermo Scientific) to obtain a cytosolic fraction and a nuclear protein fraction. Both fractions were frozen at −80°C until quantification.

### Western blotting (WB) analysis

After protein extraction from lung tissue, samples (15 µg plasmatic proteins) were electrophoresed through SDS-polyacrylamide gel and transferred to polyvinylidene difluoride membranes (Millipore). WB analysis was performed using polyclonal anti-MMP-12 (R&D Systems) and polyclonal anti-TNF-α (Sigma-Aldrich Química) antibodies and detected with a horseradish peroxidase conjugate substrate system. The blot was developed using the enhanced chemiluminescence method (Amersham Biosciences) according to the manufacturer's instructions. The membranes were re-probed with monoclonal anti-α-tubulin antibody (Sigma-Aldrich Química) to normalize for protein loading. Each band was scanned by densitometry analysis using ImageQuant software (Molecular Dynamics).

### Electrophoretic mobility shift assay (EMSA)

Commercial kit (Gel Shift Assay System, Promega) was used to detect nuclear transcription factor-κB (NF-κB) DNA binding activity. NF-κB consensus sequence was end-labeled with [γ^_32^P]-ATP, ∼6,000 Ci/mmol (GE Healthcare Bio-Sciences) and T4 polynucleotide kinase. Nuclear protein extract (5 µg) was incubated for 30 min at room temperature in binding buffer [4% glycerol, 1 mM MgCl_2_, 0,5 mM EDTA, 0,5 mM dithiothreitol (DTT), 50 mM NaCl, 10 mM Tris-HCl and 50 µg/ml poly (dI-dC)]. The appropriate amount of radiolabeled NF-κB oligonucleotide was added to each sample and the sample was incubated at room temperature for additional 20 min. Signal specificity was ensured by competition reactions using a 50-fold excess of non radiolabeled NF-κB oligonucleotide, 10 min before the addition of the radiolabeled probe. Protein-DNA complexes were subsequently resolved in a 4% acrylamide gel in 0.5× TBE as running buffer. Gels were dried and exposed to autoradiographic film at −80°C. Each band was scanned by densitometry analysis using ImageQuant software (Molecular Dynamics).

### Statistical analyses

Data are presented as means ± SEM. *P*-values of <0.05 were considered statistically significant. For time-course studies, a non parametric (Mann-Whitney U-test) method was performed to compare all CS-exposed animals to their corresponding time-matched sham-exposed controls, followed by Monte Carlo's exact methods within each set of comparisons, using the Statistical Package for the Social Science (SPSS, Inc.) software.

## Results

### COHb levels in the blood of mice after CS exposure

Blood carboxyhemoglobin levels of CS-exposed mice reached a non toxic level of ∼15.3±0.6% in C57BL/6J and ∼13.2±1% in 129S2/SvHsd mice (no differences between strains), confirming the correct exposure to tobacco smoke [29].

### Time-course changes in BAL fluid inflammatory cell profile over acute CS exposure

**Figure 1:**
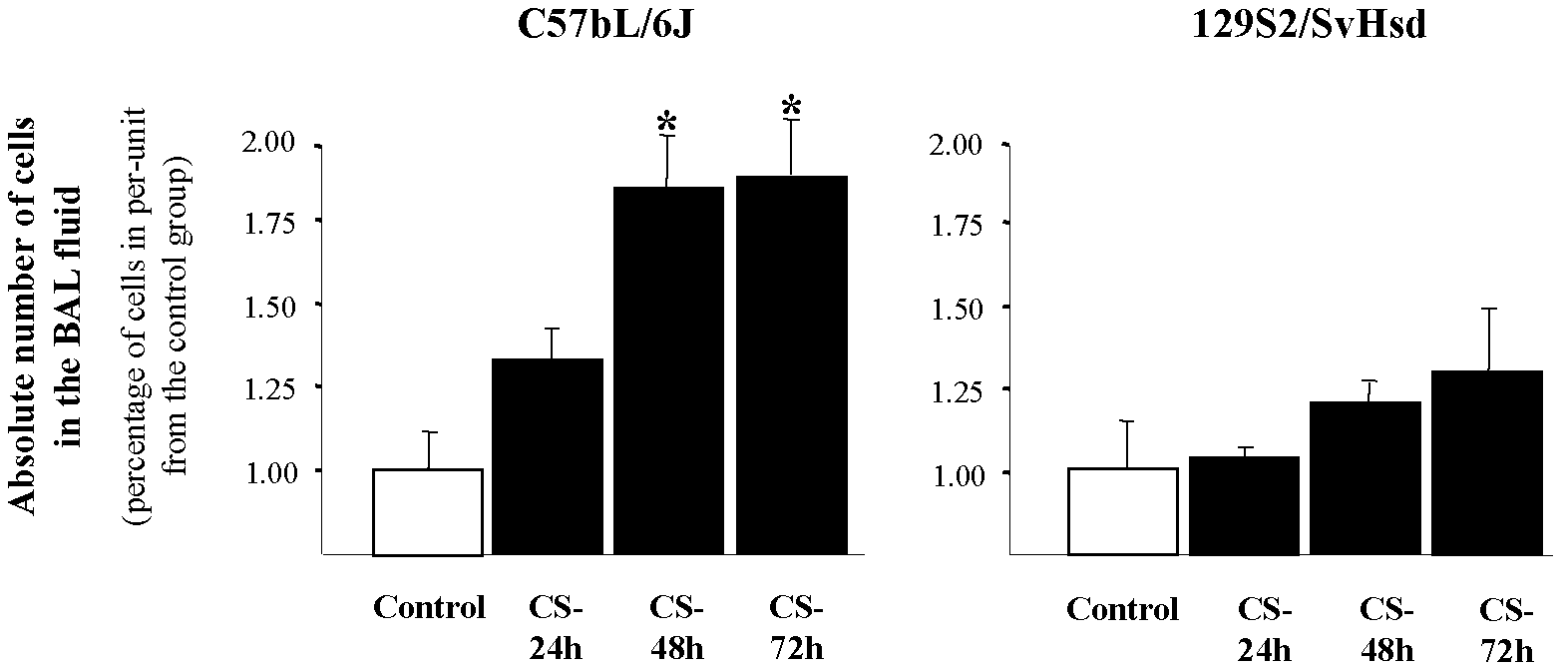
Effect of CS exposure on the absolute number of cells in BAL fluid. Increased total number of cells after CS-exposure in BAL fluid of susceptible mouse strain was found. Total cells in BAL fluid from smoke-exposed group (▪) at 24, 48 and 72 h after CS exposure in C57BL/6J (susceptible) and 129S2/SvHsd (non susceptible) mice compared to those in the air exposed group (□) and expressed the mean ± SEM; *p<0.05; n = 8/group.

In BAL fluid of CS-exposed mice, an increased total number of cells were observed as compared to air exposed mice, although this increase is only significant in C57BL/6J-susceptible mice, at 48 h (1.83±0.25) and 72 h (1.87±0.27) compared with control group (1±0.18). However, there were no significant differences in non susceptible 129S2/SvHsd mice (**Fig. 1**).

**Figure 2:**
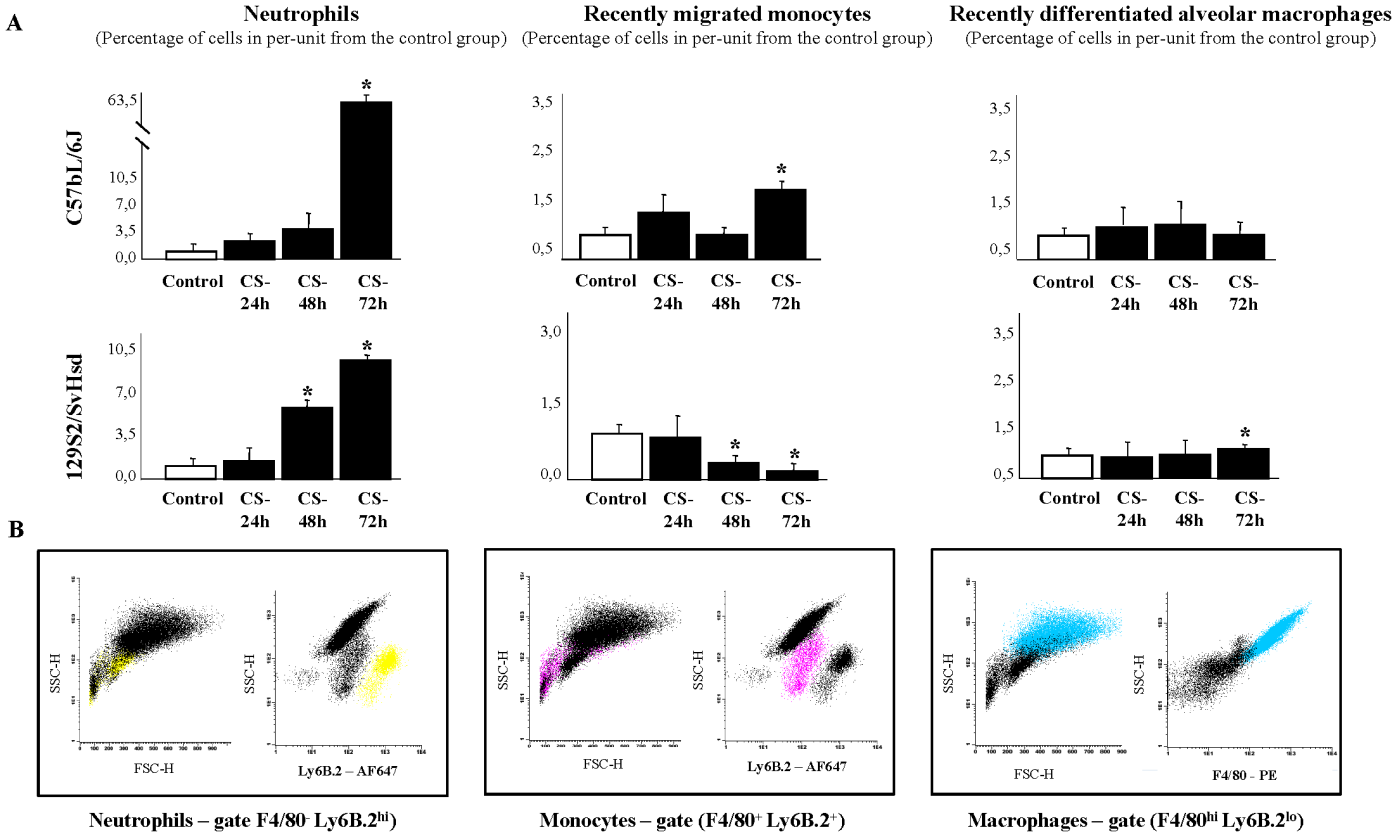
Time-course leukocytes population profile in BAL fluid after CS exposure. (**A**) Percentage of neutrophils, recently migrated monocytes and recently differentiated alveolar macrophages in BAL fluid from smoke-exposed group (▪) at 24, 48 and 72 h after CS exposure in C57BL/6J susceptible and 129S2/SvHsd non susceptible mice compared to those in the air exposed group (□) and expressed the mean ± SEM; *p<0.05; n = 8/group. (**B**) Leukocytes population was identified by flow cytometry analysis based on their characteristic properties shown in the forward scatter (FSC) and sideward scatter (SSC). Representative gating was set for Ly6B.2^hi^ on neutrophils (yellow), Ly6B.2^+^ on recently migrated monocytes (pink) and F4/80^hi^ on recently differentiated alveolar macrophages (blue) from BAL fluid of each strain of mice.

Time-course studies of leukocyte population profile in BAL fluid of mice with acute CS-induced inflammation by flow cytometry analysis were quantified. 72 h after CS exposure, our results show a significant increase in the percentage of neutrophils (63.11±0.98) and proinflammatory recently migrated monocytes (1.61±0.08) in BAL fluid of CS-exposed C57BL/6J mice, in per-unit terms with respect to the control group (1±0.06, 1±0.08 and 1±0.10, respectively). However, significant changes in the percentage of recently differentiated alveolar macrophages are not apparent in C57BL/6J mice in any time (**Fig. 2A**). In 129S2/SvHsd mice, the significant increase of neutrophils in BAL fluid occurs sooner, though not as intense as in the susceptible strain, at 48 h (4.58±1.02) and at 72 h (8.35±0.29) after CS exposure, in per-unit with respect to the air exposed group (1±0.01). Unlike susceptible mice, in 129S2/SvHsd mice a significant progressive decrease is seen in percentage of recently migrated monocytes at 48 h (0.52±0.02) and 72 h (0.48±0.04), in per-unit terms with respect to the control group (1±0.06). Moreover, there are significant differences in the percentage of recently differentiated alveolar macrophages at 72 h (1.12±0.02), in per-unit terms with respect to the control group (1±0.08) in 129S2/SvHsd mice (**Fig. 2A**). Leukocytes population was identified by flow cytometry analysis based on their characteristic properties shown in the forward scatter (FSC) and sideward scatter (SSC). Representative gating was set for Ly6B.2^hi^ on neutrophils, Ly6B.2^+^ on recently migrated monocytes and F4/80^hi^ on recently differentiated alveolar macrophages from BAL fluid of each strain of mice (**Fig. 2B**).

### MMP-12, TNF-α and NF-κB time-course response in acute CS-induced inflammation

**Figure 3:**
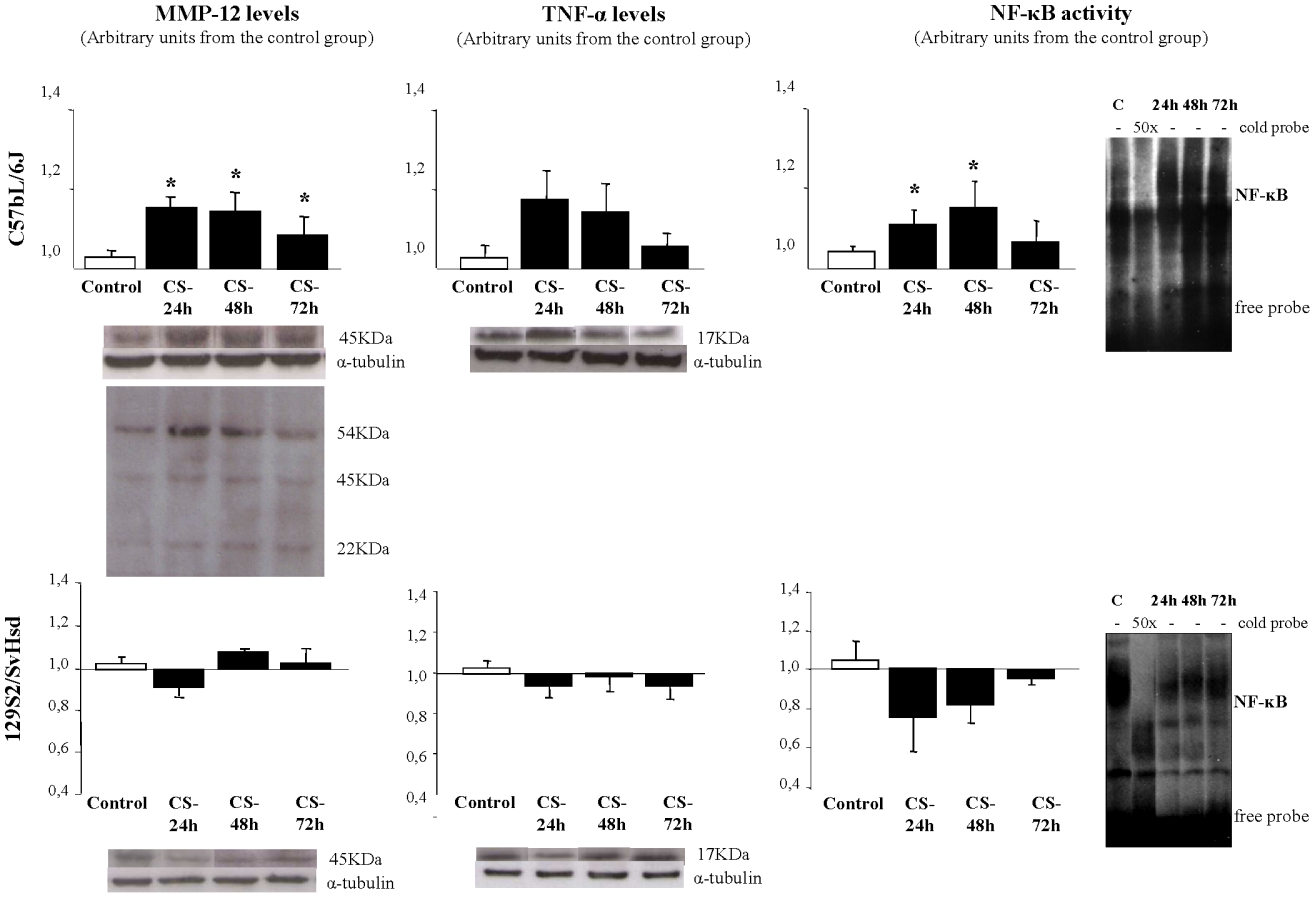
MMP-12, TNF-α levels and NF-kB activation after CS-induced inflammation. Detection of MMP-12 and TNF-α protein levels by WB and NF-kB activation by EMSA in lung homogenates after CS exposure was performed. Specificity of the bands was confirmed by the disappearance of the bands with the addition of 50-fold excess of cold oligonucleotide (cold probe). Bands were measured by densitometric analysis and normalized with α-tubulin for WB. Results were expressed as mean ± SEM; *p<0.05; n = 8/group. (□) air exposed group; (▪) smoke-exposed group at 24, 48 and 72 h after CS exposure.

Western blotting analysis showed that MMP-12 protein levels were significantly increased in C57BL/6J at 24 h (1.16±0.05), at 48 h (1.14±0.07) and at 72 h after CS exposure (1.10±0.03) relative to the control group (1±0.05), in per-unit terms. However, there was no significant difference in 129S2/SvHsd mice (**Fig. 3**). In susceptible mouse strain (C57BL/6J), TNF-α level tends to increase, but there were no significant differences between smoke-exposed and air-exposed groups. However, in non susceptible mouse strain (129S2/SvHsd), TNF-α level tends to decrease, although there were no significant differences (**Fig. 3**). Regarding to the role of NF-κB DNA binding activity, measured by electrophoretic mobility shift assay in C57BL/6J mice there was an increase 24 h (1.1±0.04), 48 h (1.18±0.04) and 72 h after CS exposure related with the control group (1±0.03). In contrast, there were no significant differences on NF-κB DNA binding activity between CS-exposed and control groups in 129S2/SvHsd although there was a tendency to decrease (**Fig. 3**).

### Confirmation that macrophage depletion in BAL fluid induced by CL_2_MDP is effective

**Figure 4:**
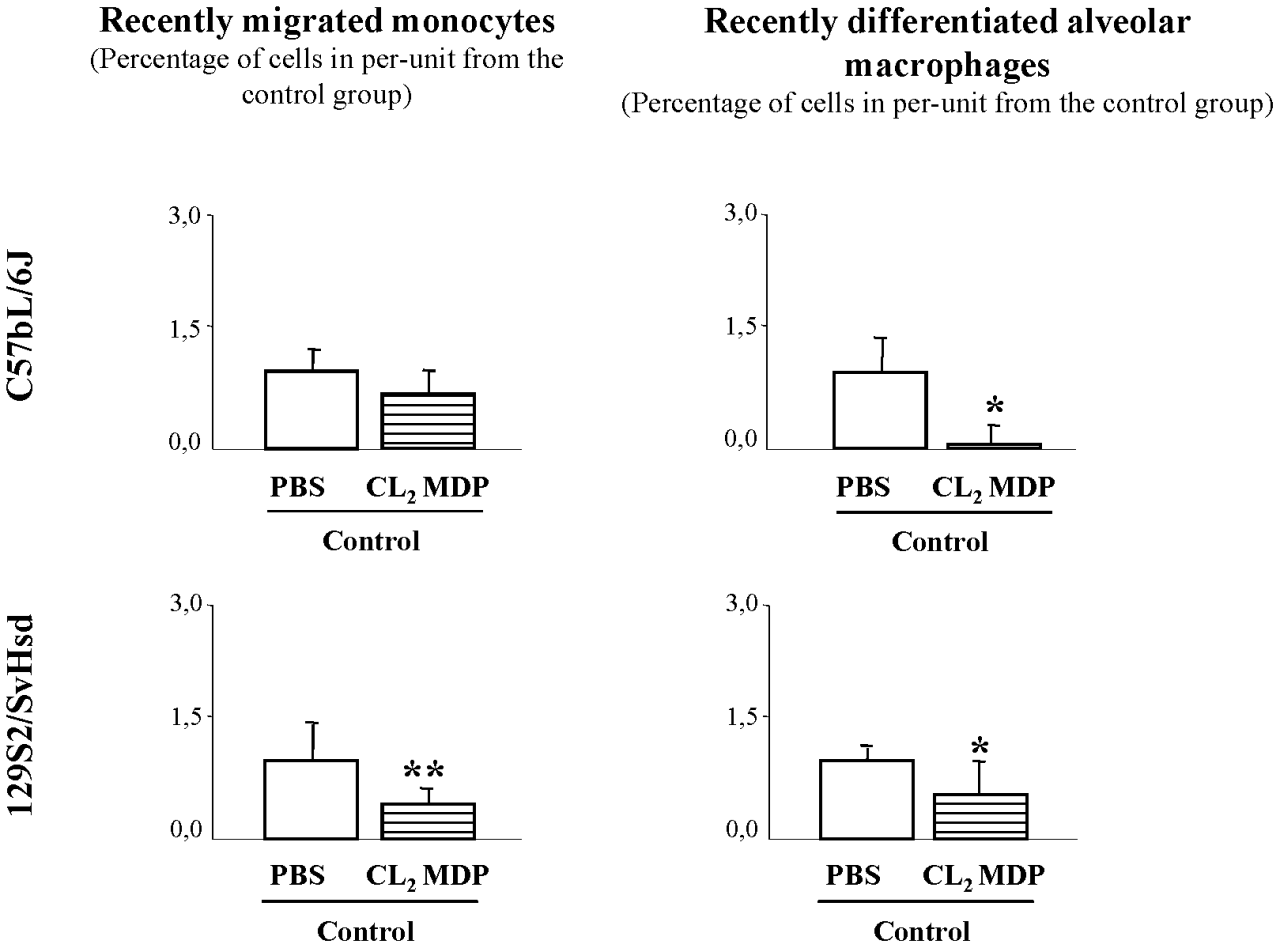
Confirmation that macrophage depletion in BAL fluid induced by CL_2_MDP is effective. Effect on macrophage depletion after CL_2_MDP treatment was studied by flow cytometry analysis in BAL fluid from C57BL/6J-susceptible and 129S2/SvHsd-resistant non-smoke-exposed mice (control group). Percentage of recently migrated monocytes and recently differentiated alveolar macrophages in BAL fluid from CL_2_MDP-treated control group (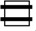) at 48 h after treatment compared to those in the PBS-treated control group (□) and expressed the mean ± SEM; *p<0.05, **p<0.01; n = 7–8/group.

When mice were examined by flow cytometry analysis for the confirmation of monocytes/macrophages depletion in BAL fluid after first liposome-encapsulated CL_2_MDP administration, it was found in C57BL/6J mice that this treatment resulted in a 49% and 67% reduction respectively at 48 h after treatment (no figure). Consequently, additional liposome-encapsulated CL_2_MDP treatment was given 48 h after first treatment and the alveolar macrophages were significantly reduced in per-unit by 90% (0.09±0.02) and monocytes by 39% (0.72±0.30) in C57BL/6J-suceptible mice compared to animals treated with liposome-encapsulated PBS in per-unit (1±0.36 and 1±0.48, respectively). In 129S2/SvHsd-non-susceptible mice alveolar macrophages were significantly reduced in per-unit by 44% (0.56±0.39) and monocytes by 56% (0.44±0.15) compared to animals treated with liposome-encapsulated PBS in per-unit (1±0.16 and 1±0.44, respectively) (**Fig. 4**).

### Repopulation profile induced by CS exposure after transient monocyte/macrophage depletion

**Figure 5:**
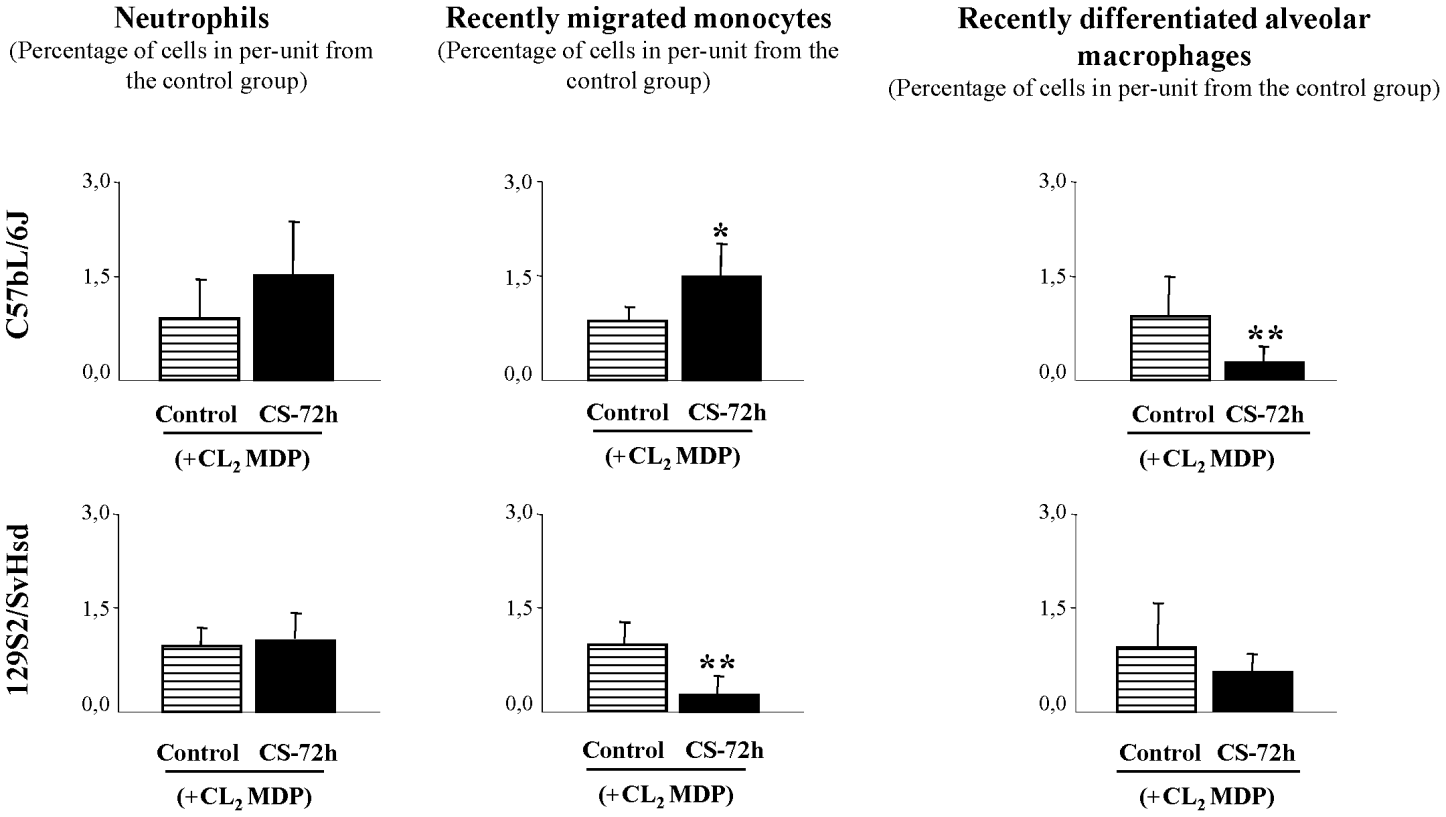
Leukocytes repopulation profile in BAL fluid induced by CS exposure after transient monocyte/macrophage. Effect of CS exposure on cell repopulation after CL_2_MDP treatment was studied by flow cytometry analysis in BAL fluid from susceptible mice. Percentage of neutrophils, recently migrated monocytes and recently differentiated alveolar macrophages in BAL fluid from smoke-exposed group (▪) at 72 h after CS exposure and pretreatment with CL_2_MDP in C57BL/6J-susceptible and 129S2/SvHsd-non susceptible mice compared with their respective CL_2_MDP -treated air exposed group (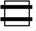) and expressed the mean ± SEM; *p<0.05, **p<0.01; n = 8/group.

To study whether the elimination of alveolar macrophages from the lung and monocytes from the blood would influence the pulmonary immune response, we expose the animals to CS exposure, 2d after last liposome doses. After Cl_2_MDP-encapsulated liposomes treatment, in C57BL/6J mice CS exposure resulted in a significant increase in recently migrated monocytes counts (1.54±0.05) in BAL fluid 72 h after CS exposure related to the control group in per-unit (1±0.03) and very significant decrease in the percentage of recently differentiated alveolar macrophages (0.34±0.01), related to the control group in per-unit (1±0.10) (**Fig. 5**). However, in 129S2/SvHsd mice after Cl_2_MDP-encapsulated liposomes treatment, CS exposure resulted in a very significant decrease in recently migrated monocytes counts (0.25±0.15) in BAL fluid 72 h after CS exposure related to the control group in per-unit (1±0.34) (**Fig. 5**).

### MMP-12 levels induced by CS exposure after transient monocyte/macrophage depletion

**Figure 6:**
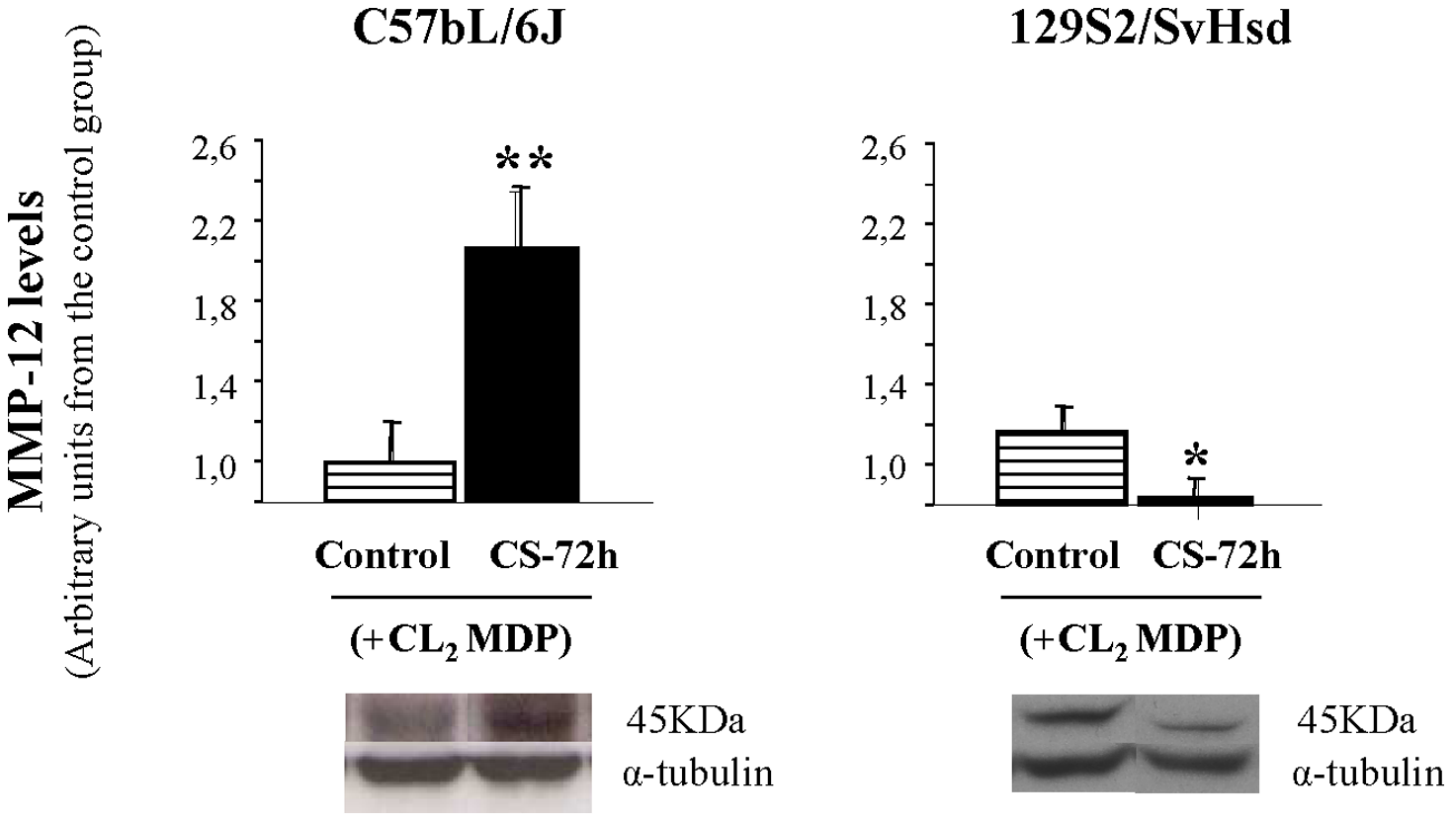
MMP-12 levels in CS-induced inflammation after CL_2_MDP treatment. Detection of MMP-12 protein levels by WB in the lung homogenates after CS exposure and pretreatment with CL_2_MDP were performed in C57BL/6J-susceptible and 129S2/SvHsd-non susceptible mice. Bands were measured by densitometric analysis and normalized with α-tubulin. Results were expressed as mean ± SEM; *p<0.05, **p<0.01; n = 6/group. (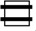) air exposed group after pretreatment with CL_2_MDP; (▪) smoke-exposed group at 72 h after CS exposure and pretreatment with CL_2_MDP.

Densitometric analysis of WB showed that MMP-12 protein levels in lung tissue homogenates by acute CS exposure after liposome-encapsulated CL_2_MDP treatment were very significantly increased in CS exposure group from C57BL/6J mice (2.14±0.08) related to the control group (1±0.02), in per-unit terms (**Fig. 6**). However, in 129S2/SvHsd mice after Cl_2_MDP-encapsulated liposomes treatment, CS exposure resulted in a significant decrease in MMP-12 protein levels in CS exposure group at 72 h (0.64±0.08) related to the control group in per-unit (1±0.13) (**Fig. 6**).

## Discussion

The effect of CS exposure in mice is believed to be strain-dependent, since inbred mouse strains can be either susceptible or resistant to the CS-induced lesions. In this regard, it has been previously reported that 129S2/SvHsd mice are resistant to the effects of smoke exposure, whereas C57BL/6J are susceptible and respond with the development of emphysema [1], [2], [3], [4], [6]. However, the cellular mechanisms responsible for the different susceptibility of these mouse strains to CS are still unknown. Identification of sensitive and resistant mouse strains could be useful for understanding the molecular mechanisms of CS effects on inflammation and pharmacological interventional studies in CS-exposure mouse models.

It is known that inflammatory cells within the lung differ in number, composition and functional properties at different stages of inflammation [30]. Macrophages are the most abundant cell type recovered from BAL fluid of animal models of emphysema induced by CS exposure [4], [31] and patients with COPD [32]. Distinct macrophage subpopulations characterize acute inflammatory lung disease [33] and macrophage number in BAL fluid correlates with disease severity [34]. However, it is still difficult to examine whether functional changes have occurred due to infiltration of cells or as a result of activation of cells which are already present. This also applies for alveolar macrophages that are present in the alveolar space at considerable numbers but may also be newly recruited from circulating monocytes during an inflammatory reaction [7], [8], [10], [12]. It has long been known that blood monocytes through the pulmonary circulation can become sequestered within pulmonary capillaries across the endothelium and migrate into the interstitial and alveolar spaces where they mature into alveolar macrophages (first into recently differentiated alveolar macrophages or parenchymal lung macrophages) [9], [35]. Moreover, alveolar macrophages are derived from recruited monocytes and not directly from the bone marrow cells as initially speculated [36]. In this study we used a specific antibody that was reported initially as “neutrophils-specific” antibody Ly6B.2 (clone 7/4) [37]. More recently, it has been observed that the 7/4-antigen is expressed on murine monocytes [38], [39], [40] and seemingly lost during differentiation to mature tissue-resident macrophages in inflammatory lesions. Identification of murine proinflammatory monocytes and understanding the mechanism of monocytes recruitment from the blood will aid further attempts to control these cells in the inflammatory conditions in which they have a central role.

To determine the functional importance of monocyte derived cells in mediating acute CS-induced airway inflammation, alveolar macrophages and blood monocytes were selectively and transiently depleted by liposome-encapsulated CL_2_MDP administration [35]. This technique depletes monocytes/macrophages without damaging other cell types in the lungs. Using this method, we were able to transiently remove alveolar macrophages from the alveolar spaces and circulating monocytes from the blood. The repopulation cellular profile in BAL fluid after CS exposure in C57BL/6J mice treated previously with CL_2_MDP, gives evidence of a large recruitment of recently migrated monocytes at 72 h after CS exposure accompanied by an increase in MMP-12 enzyme associated with tissue breakdown and a significantly decrease in the number of macrophages. While we found in 129S2/SvHsd mice a decrease in both populations recently migrated monocytes as macrophages, accompanied by decrease in the levels of MMP-12.

Our results confirm that the inflammatory phenotype differs among susceptible and non-susceptible mouse strain. Acute CS exposure encourages the recruitment of proinflammatory monocytes, promotes the release of MMP-12 and leads to robust neutrophilic inflammation at 72 h after one single exposure to three consecutive cigarettes in C57BL/6J mice. In contrast, 129S2/SvHsd mice shows decreased influx of proinflammatory monocytes, decreased levels of MMP-12 in lungs, and not so robust neutrophilic inflammation. In a recent study performed in C57BL/6J mice acutely exposed to CS, it has been proposed that MMP-12 functions also as a TNF-α-converting enzyme [41]. Our data show that MMP-2 and TNF-α level in C57BL/6J lung tissue was significantly higher than in 129S2/SvHsd after CS exposure consistently with previous *in vivo* studies [3], [6], [15], [42]. It is suggested that the involvement of MMPs in CS-induced lung inflammation is mediated by the release of TNF-α from macrophages with the subsequent neutrophil influx [41]. However, it should be noted that while TNF-α has been shown by some to be a key initiator of inflammation following CS exposure [16], we did not observe changes in TNF-α 72 h later of exposition to tobacco smoke. This is because TNF-α exhibits a peak at 2–4 h after smoke exposure, that at 6 hours returns to baseline levels [6], [15], so it is not surprising that we did not observe changes in TNF-α in these mice. Markedly higher levels of NF-κB DNA binding activity measured by electrophoretic mobility shift assay were observed only in C57BL/6J lung tissue after CS exposure. The present results are consistent with *in vivo* studies showing increased levels of NF-κB DNA binding activity in lungs of C57BL/6J and other susceptible strains in response to CS exposure being postulated that NF-κB could be one of the genetic determinants that contribute to the increased susceptibility of C57BL/6J mice to CS [3], [4].

In conclusion, we demonstrate that recently migrated monocytes into the lungs could determine the gravity of the damage caused by tobacco smoke. Our data support the impact of recently migrated monocytes recruitment as a possible marker of susceptibility to cigarette smoke-induced lung injury. Therefore the results of these experiments demonstrate that the induction of MMP-12 and the recruitment of recently migrated monocytes have an important association in the pathogenesis of susceptibility to acute pulmonary inflammation. Early diagnosis is of key importance in the treatment of emphysema. However, it would be necessary further studies to elucidate the relation between the influx of monocytes into the lungs and the activation of the inflammatory response.

## References

[1] Cavarra E, Bartalesi B, Lucattelli M, Fineschi S, Lunghi B, et al (2001) Effects of cigarette smoke in mice with different levels of alpha(1)-proteinase inhibitor and sensitivity to oxidants. Am J Respir Crit Care Med 164: 886–890. 11549550 10.1164/ajrccm.164.5.2010032

[2] Guerassimov A, Hoshino Y, Takubo Y, Turcotte A, Yamamoto M, et al (2004) The development of emphysema in cigarette smoke-exposed mice is strain dependent. Am J Respir Crit Care Med 170: 974–980. 15282203 10.1164/rccm.200309-1270OC

[3] Vecchio D, Arezzini B, Pecorelli A, Valacchi G, Martorana PA, et al Reactivity of mouse alveolar macrophages to cigarette smoke is strain dependent. Am J Physiol Lung Cell Mol Physiol 298: L704–713. 20154225 10.1152/ajplung.00013.2009

[4] Yao H, Edirisinghe I, Rajendrasozhan S, Yang SR, Caito S, et al (2008) Cigarette smoke-mediated inflammatory and oxidative responses are strain-dependent in mice. Am J Physiol Lung Cell Mol Physiol 294: L1174–1186. 18375740 10.1152/ajplung.00439.2007

[5] Morris A, Kinnear G, Wan WY, Wyss D, Bahra P, et al (2008) Comparison of cigarette smoke-induced acute inflammation in multiple strains of mice and the effect of a matrix metalloproteinase inhibitor on these responses. J Pharmacol Exp Ther 327: 851–862. 18806126 10.1124/jpet.108.140848

[6] Churg A, Dai J, Tai H, Xie C, Wright JL (2002) Tumor necrosis factor-alpha is central to acute cigarette smoke-induced inflammation and connective tissue breakdown. Am J Respir Crit Care Med 166: 849–854. 12231496 10.1164/rccm.200202-097OC

[7] Maus U, Huwe J, Ermert L, Ermert M, Seeger W, et al (2002) Molecular pathways of monocyte emigration into the alveolar air space of intact mice. Am J Respir Crit Care Med 165: 95–100. 11779737 10.1164/ajrccm.165.1.2106148

[8] Geissmann F, Jung S, Littman DR (2003) Blood monocytes consist of two principal subsets with distinct migratory properties. Immunity 19: 71–82. 12871640 10.1016/s1074-7613(03)00174-2

[9] Landsman L, Varol C, Jung S (2007) Distinct differentiation potential of blood monocyte subsets in the lung. J Immunol 178: 2000–2007. 17277103 10.4049/jimmunol.178.4.2000

[10] Maus UA, Janzen S, Wall G, Srivastava M, Blackwell TS, et al (2006) Resident alveolar macrophages are replaced by recruited monocytes in response to endotoxin-induced lung inflammation. Am J Respir Cell Mol Biol 35: 227–235. 16543608 10.1165/rcmb.2005-0241OC

[11] Sunderkotter C, Nikolic T, Dillon MJ, Van Rooijen N, Stehling M, et al (2004) Subpopulations of mouse blood monocytes differ in maturation stage and inflammatory response. J Immunol 172: 4410–4417. 15034056 10.4049/jimmunol.172.7.4410

[12] Muller WA (2001) New mechanisms and pathways for monocyte recruitment. J Exp Med 194: F47–51. 11696603 10.1084/jem.194.9.f47 PMC2195978

[13] Barnes PJ (2004) Alveolar macrophages as orchestrators of COPD. COPD 1: 59–70. 16997739 10.1081/COPD-120028701

[14] Demedts IK, Morel-Montero A, Lebecque S, Pacheco Y, Cataldo D, et al (2006) Elevated MMP-12 protein levels in induced sputum from patients with COPD. Thorax 61: 196–201. 16308335 10.1136/thx.2005.042432 PMC2080750

[15] Churg A, Wang RD, Tai H, Wang X, Xie C, et al (2003) Macrophage metalloelastase mediates acute cigarette smoke-induced inflammation via tumor necrosis factor-alpha release. Am J Respir Crit Care Med 167: 1083–1089. 12522030 10.1164/rccm.200212-1396OC

[16] Churg A, Wang RD, Tai H, Wang X, Xie C, et al (2004) Tumor necrosis factor-alpha drives 70% of cigarette smoke-induced emphysema in the mouse. Am J Respir Crit Care Med 170: 492–498. 15184206 10.1164/rccm.200404-511OC

[17] Moodie FM, Marwick JA, Anderson CS, Szulakowski P, Biswas SK, et al (2004) Oxidative stress and cigarette smoke alter chromatin remodeling but differentially regulate NF-kappaB activation and proinflammatory cytokine release in alveolar epithelial cells. FASEB J 18: 1897–1899. 15456740 10.1096/fj.04-1506fje

[18] Van Rooijen N (1989) The liposome-mediated macrophage ‘suicide’ technique. J Immunol Methods 124: 1–6. 2530286 10.1016/0022-1759(89)90178-6

[19] Claassen I, Van Rooijen N, Claassen E (1990) A new method for removal of mononuclear phagocytes from heterogeneous cell populations in vitro, using the liposome-mediated macrophage ‘suicide’ technique. J Immunol Methods 134: 153–161. 2147710 10.1016/0022-1759(90)90376-7

[20] van Rooijen N, Bakker J, Sanders A (1997) Transient suppression of macrophage functions by liposome-encapsulated drugs. Trends Biotechnol 15: 178–185. 9161052 10.1016/s0167-7799(97)01019-6

[21] Van Rooijen N, Sanders A (1994) Liposome mediated depletion of macrophages: mechanism of action, preparation of liposomes and applications. J Immunol Methods 174: 83–93. 8083541 10.1016/0022-1759(94)90012-4

[22] Sekhon HS, Wright JL, Churg A (1994) Cigarette smoke causes rapid cell proliferation in small airways and associated pulmonary arteries. Am J Physiol 267: L557–563. 7977766 10.1152/ajplung.1994.267.5.L557

[23] Perez-Rial S, Del Puerto-Nevado L, Gonzalez-Mangado N, Peces-Barba G (2011) Early detection of susceptibility to acute lung inflammation by molecular imaging in mice exposed to cigarette smoke. Mol Imaging 10: 398–405. 21914430 10.2310/7290.2011.00010

[24] van Rooijen N, Sanders A, van den Berg TK (1996) Apoptosis of macrophages induced by liposome-mediated intracellular delivery of clodronate and propamidine. J Immunol Methods 193: 93–99. 8690935 10.1016/0022-1759(96)00056-7

[25] van Rooijen N, van Kesteren-Hendrikx E (2003) “In vivo” depletion of macrophages by liposome-mediated “suicide”. Methods Enzymol 373: 3–16. 14714393 10.1016/s0076-6879(03)73001-8

[26] Van Rooijen N, Kors N, vd Ende M, Dijkstra CD (1990) Depletion and repopulation of macrophages in spleen and liver of rat after intravenous treatment with liposome-encapsulated dichloromethylene diphosphonate. Cell Tissue Res 260: 215–222. 2141546 10.1007/BF00318625

[27] Thepen T, Van Rooijen N, Kraal G (1989) Alveolar macrophage elimination in vivo is associated with an increase in pulmonary immune response in mice. J Exp Med 170: 499–509. 2526847 10.1084/jem.170.2.499 PMC2189410

[28] Wilson MR, O'Dea KP, Zhang D, Shearman AD, van Rooijen N, et al (2009) Role of lung-marginated monocytes in an in vivo mouse model of ventilator-induced lung injury. Am J Respir Crit Care Med 179: 914–922. 19218195 10.1164/rccm.200806-877OC PMC2684018

[29] Sen S, Peltz C, Beard J, Zeno B (2010) Recurrent carbon monoxide poisoning from cigarette smoking. Am J Med Sci 340: 427–428. 20739872 10.1097/MAJ.0b013e3181ef712d

[30] Lofdahl JM, Wahlstrom J, Skold CM (2006) Different inflammatory cell pattern and macrophage phenotype in chronic obstructive pulmonary disease patients, smokers and non-smokers. Clin Exp Immunol 145: 428–437. 16907910 10.1111/j.1365-2249.2006.03154.x PMC1809704

[31] Churg A, Zay K, Shay S, Xie C, Shapiro SD, et al (2002) Acute cigarette smoke-induced connective tissue breakdown requires both neutrophils and macrophage metalloelastase in mice. Am J Respir Cell Mol Biol 27: 368–374. 12204900 10.1165/rcmb.4791

[32] Tetley TD (2002) Macrophages and the pathogenesis of COPD. Chest 121: 156S–159S. 12010845 10.1378/chest.121.5_suppl.156s

[33] Duan M, Li WC, Vlahos R, Maxwell MJ, Anderson GP, et al Distinct macrophage subpopulations characterize acute infection and chronic inflammatory lung disease. J Immunol 189: 946–955. 22689883 10.4049/jimmunol.1200660

[34] Hogg JC, Chu F, Utokaparch S, Woods R, Elliott WM, et al (2004) The nature of small-airway obstruction in chronic obstructive pulmonary disease. N Engl J Med 350: 2645–2653. 15215480 10.1056/NEJMoa032158

[35] Landsman L, Jung S (2007) Lung macrophages serve as obligatory intermediate between blood monocytes and alveolar macrophages. J Immunol 179: 3488–3494. 17785782 10.4049/jimmunol.179.6.3488

[36] Murphy J, Summer R, Wilson AA, Kotton DN, Fine A (2008) The prolonged life-span of alveolar macrophages. Am J Respir Cell Mol Biol 38: 380–385. 18192503 10.1165/rcmb.2007-0224RC PMC2274942

[37] Hirsch S, Gordon S (1983) Polymorphic expression of a neutrophil differentiation antigen revealed by monoclonal antibody 7/4. Immunogenetics 18: 229–239. 6618532 10.1007/BF00952962

[38] Henderson RB, Hobbs JA, Mathies M, Hogg N (2003) Rapid recruitment of inflammatory monocytes is independent of neutrophil migration. Blood 102: 328–335. 12623845 10.1182/blood-2002-10-3228

[39] Rosas M, Thomas B, Stacey M, Gordon S, Taylor PR (2010) The myeloid 7/4-antigen defines recently generated inflammatory macrophages and is synonymous with Ly-6B. J Leukoc Biol 88: 169–180. 20400676 10.1189/jlb.0809548 PMC2892525

[40] Taylor PR, Brown GD, Geldhof AB, Martinez-Pomares L, Gordon S (2003) Pattern recognition receptors and differentiation antigens define murine myeloid cell heterogeneity ex vivo. Eur J Immunol 33: 2090–2097. 12884282 10.1002/eji.200324003

[41] Sato A, Hoshino Y, Hara T, Muro S, Nakamura H, et al (2008) Thioredoxin-1 ameliorates cigarette smoke-induced lung inflammation and emphysema in mice. J Pharmacol Exp Ther 325: 380–388. 18256171 10.1124/jpet.107.134007

[42] Hautamaki RD, Kobayashi DK, Senior RM, Shapiro SD (1997) Requirement for macrophage elastase for cigarette smoke-induced emphysema in mice. Science 277: 2002–2004. 9302297 10.1126/science.277.5334.2002

